# Storage conditions and passages alter IL-6 secretion in C26 adenocarcinoma cell lines

**DOI:** 10.1016/j.mex.2015.02.001

**Published:** 2015-02-07

**Authors:** Diana M. Norden, Raymond Devine, Donna O. McCarthy, Loren E. Wold

**Affiliations:** a Department of Neuroscience, The Ohio State University, 333 W. 10th Ave, Columbus, OH, United States; b Davis Heart and Lung Research Institute, 473 W. 12th Ave, The Ohio State University, Columbus, OH, United States; c College of Nursing, Marquette University, Milwaukee, WI, United States; d College of Nursing, The Ohio State University, 1585 Neil Ave, Columbus, OH, United States

**Keywords:** Cancer cachexia cell line maintenance, Cancer cachexia, Tumor cell line, IL-6

## Abstract

The C26 adenocarcinoma tumor line is frequently used to establish peripheral tumors in mice for the study of cancer cachexia and cancer-related fatigue. Recently, we have noticed a progressive decline in the effects of tumor growth on our biological and behavioral measures in the tumor-bearing mice. Therefore, we compared effects of three aliquots of the C26 tumor cell line that differed in storage condition and number of passages on cytokine secretion, tumor growth, weight loss and fatigue behavior. Three aliquots of the C26 tumor line were selected as alpha (α), beta (β), and gamma (γ). Aliquot α was an original C26 stock line that had been stored at −80 °C. Aliquot β was stored in liquid nitrogen. Aliquot γ was taken from aliquot β and passaged three times. The three aliquots of the C26 tumor line showed differences in IL-6 mRNA and protein secretion *in vitro*, with aliquot β showing the greatest IL-6 secretion. These differences were mirrored *in vivo*. Plasma IL-6 levels were elevated in all tumor bearing mice but was greatest in group β mice. Carcass weight was decreased in all three tumor groups. Brain expression of IL-1β mRNA was greatest in group β and group β demonstrated the greatest decline in running activity at day 19.

• Storage conditions and number of passages influence C26 tumor cell secretion of cytokines.
• Variations in C26 aliquots may explain differences observed between laboratories using the same cell line.
• We recommend always storing cell lines in liquid nitrogen and limiting the number of passages before use in experiments.

## Graphical abstract

**Figure:**
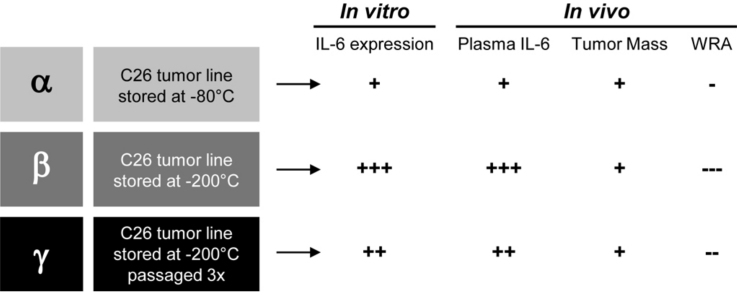

## Methods

### Cell line

Three aliquots of the colon26 adenocarcinoma (C26) cell line were thawed and maintained in culture with RPMI 1640 media supplemented with 10% FBS. The three aliquots were termed alpha (α), beta (β), and gamma (γ). The aliquots differed in storage condition and number of passages. Aliquot α was an original C26 stock line that was stored at −80 °C. Aliquot β was stored in liquid nitrogen. Aliquot γ was taken from aliquot β and passaged three times. These conditions were selected as representations of common lab practice. For example, cell lines may be temporarily stored at −80 °C during re-location and during shipping. Cell lines may also be passaged numerous times during ongoing experiments. Once confluent, cells were harvested using 0.05% trypsin–EDTA (Gibco), washed in phosphate buffered saline (PBS), counted using 0.04% trypan blue, and resuspended at 2.5 × 10^6^ cells/ml in PBS for injection [8]. An aliquot of each cell line was plated in 24 well plates at a density of 100,000 cells/well (*n* = 4). Media was replaced the following day. After 24 h, media was collected and frozen at −80 °C and RNA was isolated using a tri-reagent protocol.

### Mice

Twenty adult (10 weeks) female BALB/c × DBA/2 F1 (CD2F1) mice weighing 20–22 g were obtained from Charles River Laboratories. Mice were singly housed and maintained at 25 °C under a 12 h light cycle with *ad libitum* access to food and water. All procedures were performed in accordance with the National Institute of Health Guidelines for the Care and Use of Laboratory Animals and were approved by The Ohio State University Institutional Animal Care and Use Committee.

### Tumor injection

Groups of five mice were injected subcutaneously between the scapulae with 5 × 10^5^ cells from one of three aliquots of the C26 cell line in 0.2 ml of PBS and five were injected with PBS alone. This tumor cell line is syngeneic for CD2F1 mice and secretes IL-6 and TNF-α [3] and does not metastasize when injected subcutaneously [7]. Tumor growth is usually palpable by day 7, weight loss and muscle wasting are evident after day 14, and mice become moribund by day 24 of tumor-growth. In the present study, all data collection was completed on day 21 of tumor growth. Mice were euthanized by inhalation of CO_2_ gas on day 21 of tumor growth. Blood was withdrawn from the brachial artery. Tumors were dissected and weighed and the brain was quickly dissected and snap frozen in liquid nitrogen.

## IL-6 ELISA

IL-6 levels in the culture medium and plasma were determined using the BD OptEIA Mouse IL-6 ELISA, according to the manufacturer’s instructions (BD Biosciences). Absorbance was read at 450 nm using a Synergy HT Plate Reader (Bio-tek instruments). The assay was sensitive to 10 ng/ml IL-6 and intra-assay coefficients of variation were less than 10%.

### Voluntary wheel running activity

Fatigue was modeled as a decline in voluntary wheel running activity (VWRA) in the tumor-bearing mice [9]. Mice were singly housed and acclimated to a four inch diameter running wheel in the cage for one week, and baseline measures (week 0) of VWRA were recorded overnight prior to injection of tumor cells or PBS. Wheels were again placed in the home cages of all mice overnight (6 p.m. to 8 a.m.) on days 9 and 19 of tumor growth and the total number of turns each night was digitally recorded (Columbus Instruments, model 0297-004M).

### RNA isolation and RT-PCR analysis

RNA was isolated from cells and brain sections using the Tri-Reagent protocol (Sigma) and reverse transcribed to cDNA using the High Capacity cDNA Reverse Transcription kit (Applied Biosystems). Quantitative PCR was performed using the Applied Biosystems Assay-on-Demand Gene Expression protocol. Experimental cDNA was amplified with an ABI PRISM 7300-sequence detection system (Applied Biosystems) using real-time PCR and normalized based on reference cDNA (GAPDH). Data were analyzed with the comparative threshold cycle method. Data are expressed as fold change from control.

### Statistical analyses

To ensure a normal distribution, data were analyzed using a Shapiro–Wilk test with the Statistical Analysis Systems (SAS) statistical software (Cary, NC). To determine significance, data were analyzed using a one-way ANOVA through the General Linear Model procedures of SAS. Differences between treatment means were evaluated by an *F*-protected *t*-test using the Least-Significant Difference procedure of SAS. All data are expressed as treatment means ± standard error of the mean (SEM). Values were considered significant at *p*-values <0.05.

## Results

**Fig. 1:**
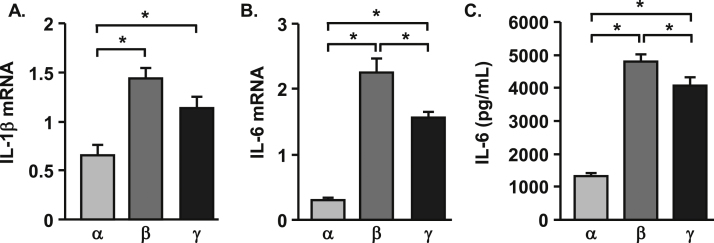
(A) IL-1β and (B) IL-6 mRNA expression in three aliquots of the C26 tumor cell line. (C) IL-6 levels in the media after 24 h. *p < 0.05 (*n* = 4).

The three different aliquots of C26 cells expressed variable amounts of IL-1β and IL-6 mRNA. All aliquots secreted IL-6 protein into the culture medium and levels of IL-6 protein were greatest in media from aliquot β (Fig. 1, *p *< 0.05). Aliquot α had the lowest levels of IL-6 mRNA and protein expression (*p *< 0.05).

**Fig. 2:**
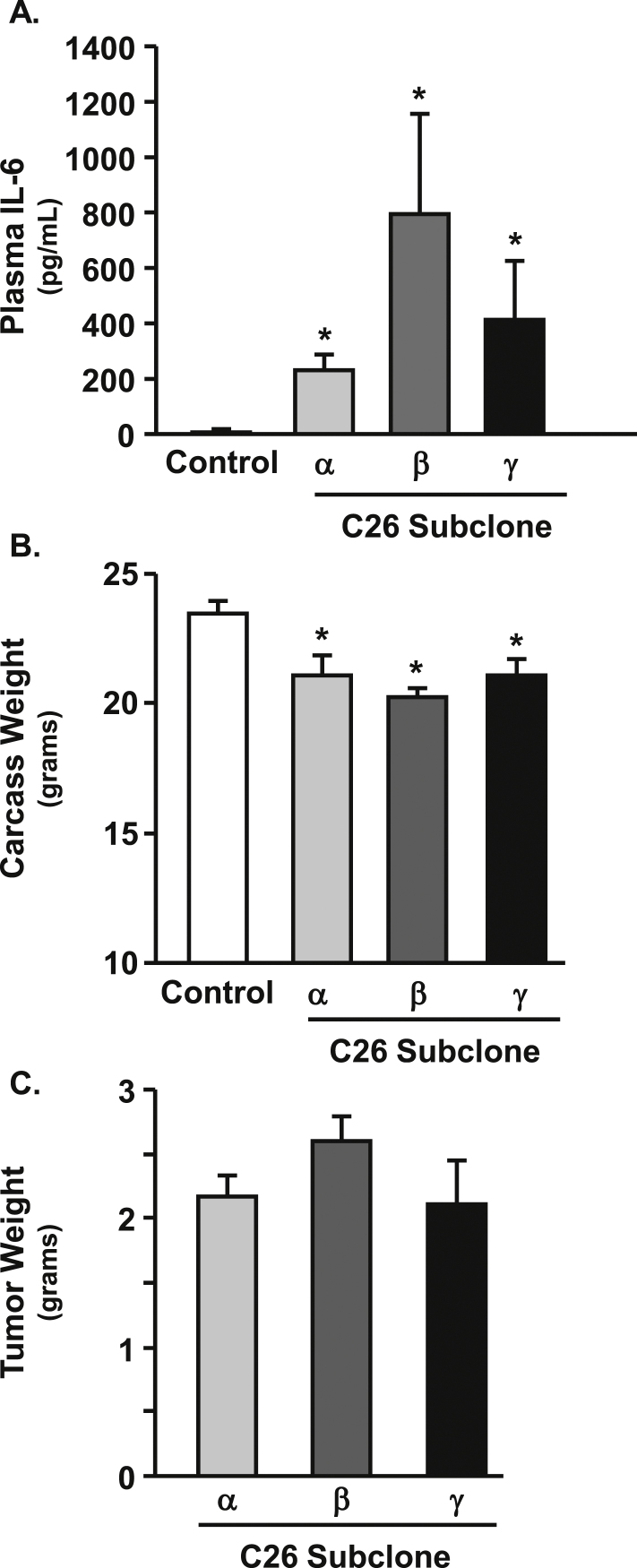
(A) Plasma IL-6 levels in control and α, β, γ tumor-bearing mice at 21 days. (B) Carcass weight at day 21. (C) Tumor mass at day 21. *p < 0.05 from control (*n* = 5).

One of the three aliquots was injected into five mice to establish a peripheral tumor. This model is commonly used in studies of cancer cachexia [1] and cancer-related fatigue [6]. Following 21 days of tumor growth, all tumor-bearing mice had elevated levels of IL-6 in the plasma compared to control animals (Fig. 2A, *p *< 0.05). Although the data may indicate that mice injected with aliquot β had the highest amount of plasma IL-6, there were no significant differences between groups, most likely due to the low number of animals used and variability between samples. All tumor-bearing mice showed signs of cachexia with decreased carcass weight by day 21(Fig. 2B, *p *< 0.05). There was no difference in tumor mass between groups (Fig. 2C).

**Fig. 3:**
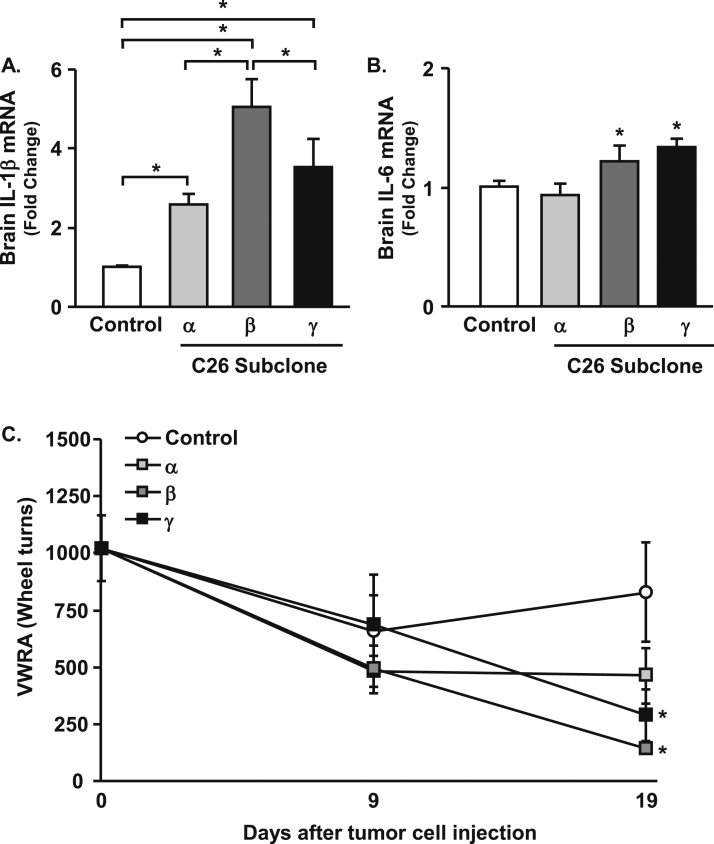
(A) IL-1β and (B) IL-6 mRNA expression in the brain of control and α, β, γ tumor bearing mice at 21 days *p < 0.05. (C) Wheel running activity of control and α, β, γ tumor bearing mice before injection and again at 9 and 19 days after injection. *p < 0.05 from control (*n* = 5).

Peripheral tumor growth and systemic inflammation increase inflammation in the brain which may influence fatigue behaviors [6]. Expression of IL-1β mRNA was elevated in the brain of all tumor-bearing mice but was highest in mice injected with aliquot β (Fig. 3A, *p *< 0.05). IL-6 expression in the brain was only increased in groups β and γ (Fig. 3B, *p *< 0.05). Fatigue, modeled as decreased VWRA, (WRA) [9] was only detected in group β and γ (Fig. 3C, *p *< 0.05).

## Additional information

In conclusion, we found that the three aliquots of the C26 tumor cell line maintained in our laboratory varied in expression of IL-6 mRNA and protein, and in development of cancer cachexia and fatigue-like behaviors in the tumor bearing mice. We found distinct and significant differences between aliquots α and β/γ, indicating that storage conditions alter the C26 phenotype. The differences between β and γ were modest, suggesting that three passages may alter C26 phenotype. However, increased passage number may show more striking differences. Overall, our data support other reports showing that aliquots of the same C26 tumor line can be used for studies of both mild and severe cachexia [2], [5]. These results may also explain variations between study findings in different laboratories that use the C26 cell line. However, the cause of this “drift” in tumor phenotype is not fully understood. One possible mechanism might be genetic or epigenetic changes in gene expression or cytogenetic/chromosomal rearrangements during freezing or passage of cells [4]. We recommend always storing cell lines for *in vivo* tumor studies in liquid nitrogen and also limiting the number of passages to increase consistency and reliability when using the C26 tumor cell line.
